# Full-field electroretinogram recorded with skin electrodes in 6- to 12-year-old children

**DOI:** 10.1007/s10633-023-09944-9

**Published:** 2023-08-02

**Authors:** Jiajun Wang, Yalan Wang, Weichen Guan, Yun-E. Zhao

**Affiliations:** 1 Eye Hospital and School of Ophthalmology and Optometry, Wenzhou Medical University, Wenzhou, Zhejiang China; 2 National Clinical Research Center for Ocular Disease, Wenzhou, Zhejiang China

**Keywords:** LA 3 ERG, Photopic negative response (PhNR), Pediatric reference data, Skin electrodes

## Abstract

**Purpose:**

To determine the full-field electroretinogram (ffERG) parameters, including the light-adapted (LA) 3 ERG and the photopic negative response (PhNR), in 6- to 12-year-old children.

**Methods:**

ffERG data were obtained from 214 eyes of 214 healthy subjects. The amplitudes and peak time of the ffERG responses were obtained from children divided into 6- to 8-year-old and 9- to 12-year-old groups. Using a skin electrode, electrical signals were measured in response to white stimulating light and white background light (LA 3 ERG). A blue background light and red flashes were then used to elicit the PhNR.

**Results:**

The a-wave amplitude ranged from 0.40 to 9.20 μV, the b-wave ranged from 4.70 to 30.80 μV, and the PhNR ranged from 1.30 to 39.90 μV. The b-wave peak time (33.20 ms) of 6- to 8-year-old groups was slightly shorter than that of the 9- to 12-year-old groups (33.60 ms, *P* = *0.01*), but no differences in amplitudes or in peak time of other components. There were significant correlations between the amplitudes (a-wave and b-wave: *r* = 0.43, *p* < 0.001; a-wave and PhNR: *r* = *0.25, p* < *0.001*; b-wave and PhNR: *r* = *0.45, p* < *0.001*). There was a moderate correlation between the a-wave and b-wave peak time (*r* = *0.31, P* < *0.001*).

**Conclusions:**

We determined the largest dataset of the LA 3 ERG and PhNR parameters in a population of healthy children, aged 6–12 years, which may provide a useful reference value when evaluating children with potential retinal defects.

## Introduction

Because light stimulation can induce retinal electrical activity, electroretinograms (ERGs) are used extensively for clinical identification of retinal diseases and evaluation of their severity [1]. Recently, the International Society of Electrophysiology of Vision (ISCEV) standard specified six recording conditions for the ffERG and an extended protocol for the photopic negative response (PhNR) [2]. These conditions were established to reflect the function of the main physiological generators located in the different retinal layers [3–5].

Currently, the negative a-wave and the positive b-wave are the main ERG components used in clinical practice. Under photopic conditions, the negative a-wave is thought to predominantly reflect activity from cone cells and OFF bipolar cells [6]. The b-wave is mainly generated by ON bipolar cells [7], with some contributions of other post-receptoral sources [8]. The PhNR is predominantly generated by retinal ganglion cells [9].

Abnormal photopic ERG components were previously reported in diseases such as retinitis pigmentosa [10], optic nerve hypoplasia [11], age-related macular degeneration [12], diabetic retinopathy [13], and aniridia [14]. A reduction in the PhNR amplitude was also described in pathological diseases such as glaucoma [15, 16], multiple sclerosis [17], childhood optic gliomas [18], and optic neuropathy [19]. Although standard ffERG values from the normal retina are required for assessment of pathological retina, these values have not been reported in a large cohort of healthy children. It also remains unclear whether the a-wave, b-wave, and PhNR amplitudes change with age especially in 6- to 12-year-old children. Furthermore, children have a preference for skin electrodes, although these typically produce lower amplitude responses than those obtained with a contact lens [20, 21]. Previous studies using skin electrodes also had relatively small sample sizes. For example, Soekamto et al. [22] recorded scotopic (rod) and photopic (cone) responses from only 20 healthy patients. Additionally, although there are some other reports of normal electrophysiological values [23–26], they are typically recorded from older subjects or do not assess the PhNR.

Therefore, the aims of this study were to record the LA 3 ERG and PhNR with a skin electrode in a large population of healthy children and to determine the relationship between these electrophysiological parameters and age.

## Subjects and methods

### Subjects

The present study included 214 eyes of 214 children with emmetropia or ametropia who volunteered to receive a full-field ERG examination at the Eye Hospital of Wenzhou Medical University at Hangzhou between September 2019 and September 2020. The inclusion criteria included: (1) age < 12 years old, (2) best-corrected visual acuity reaching the current age standard, (3) refractive error <  ± 6.00 D mean sphere, (4) a pupil diameter of ≥ 6 mm with dilation, and (5) a normal intraocular pressure. Exclusion criteria included the presence of tropia, nystagmus, fundus disease, or any physical or mental disability that could affect cooperation of the subject. We divided children into 6- to 8-year-old and 9- to 12-year-old groups for comparisons of the electrophysiological parameters.

Written informed consent was obtained from all the parents or guardians of each subject after a thorough explanation of the study. This study was approved by the Institutional Ethics Committee of Wenzhou Medical University. The study was conducted in accordance with the tenets of the Declaration of Helsinki and was registered at www.clinicaltrials.gov (NCT04427748).

### Examination

Axial length (AL) was measured using an IOL-Master 700 (Carl Zeiss Meditec AG, Jena, Germany). The manifest refraction was also assessed in all subjects.

### ERG recordings

The ffERG was performed using the Metrovision vision monitor (Metrovision, Pérenchies, France) and Ag–AgCl electrodes (EEGWO2, Brain Science Electronic&Technology Co, Qindao, China). ISCEV standard protocols for recording the LA 3 ERG and PhNR were followed as strictly as possible. The ffERG was performed binocularly on the eyes with pupils dilated using 1% tropicamide and active skin electrodes. The active electrode was taped on the skin at 2.5 mm below the margin of the lower eyelid, the earth electrode was taped at the mid-frontal position, and the reference electrode was taped at the temporal canthus position. Good electrical conduction was ensured by using an abrasive conductive gel before electrode taping. The impedances of skin electrodes were accepted be 5 kΩ or less in our study.

Video monitoring with a near-infrared sensor was used to record the eye image to ensure fixation. The LA 3 ERG condition was elicited by white flashes (3.1 cd s/m^2^) on a steady white background (31 cd/m^2^) after 10 min of light adaptation with a steady background light (31 cd/m^2^). PhNR was then elicited by red flashes (wavelength, 619 nm; 1.2 cd/m^2^) presented on a steady blue background (wavelength, 465 nm; 8 cd/m^2^). Signals were then filtered (1–35 Hz for LA 3 ERG; 1–288 Hz for PhNR), amplified (50 K), and digitized at 2 kHz. Fifty test flashes for LA 3 ERG and 200 tests for PhNR were averaged with automatic artifact rejection. The PhNR was excluded if continuous, large, or small transients were present in the waveform.

**Fig. 1:**
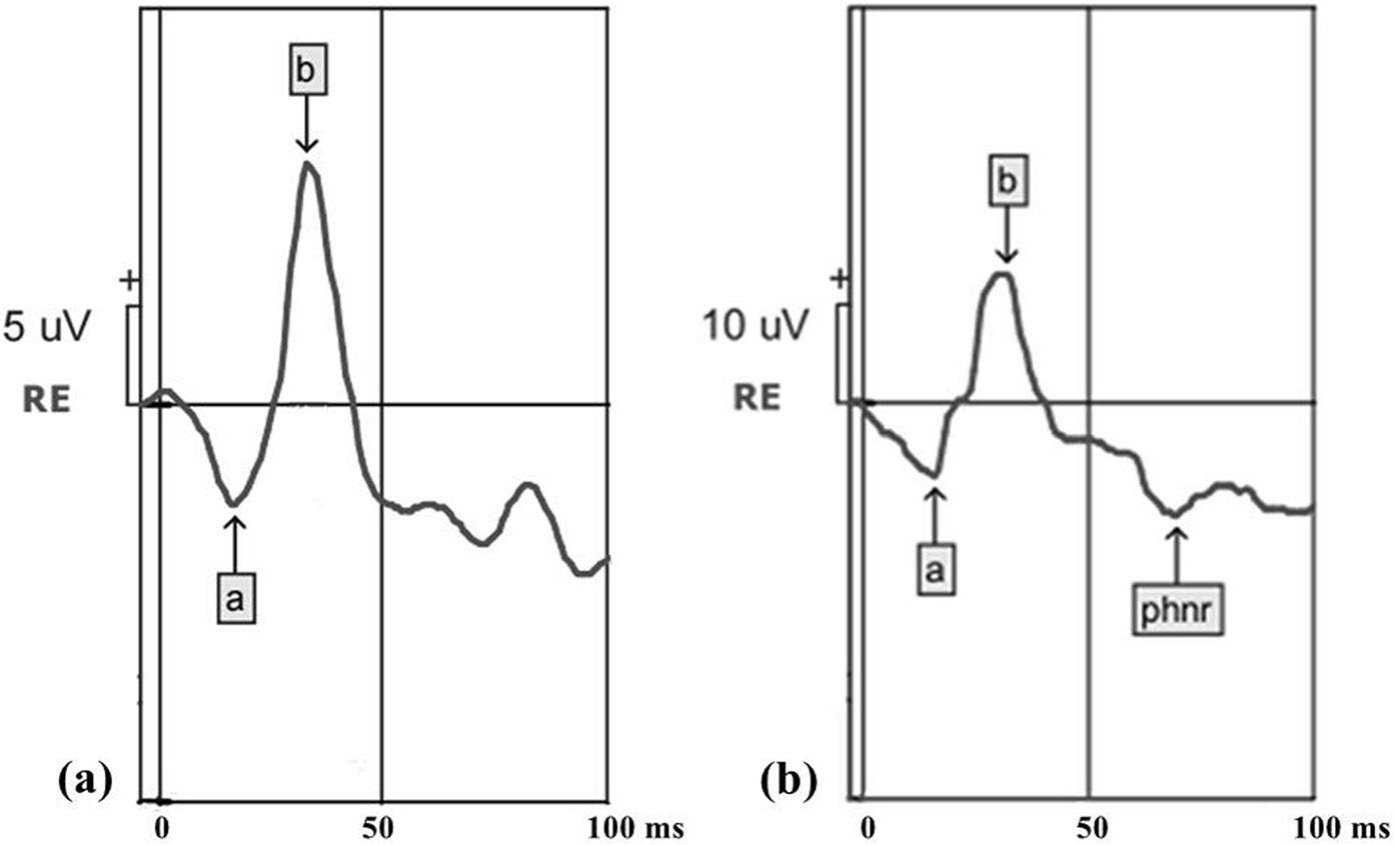
Representative waveforms of the LA 3 ERG (**a**), with the presence of a-wave and b-wave (arrows), and the photopic negative response (**b**) recordings

The a-wave and b-wave amplitudes and peak time in LA 3 ERG and the PhNR amplitude were measured. The a-wave amplitude of the LA 3 ERG was measured from baseline to its trough. The b-wave amplitude of the LA 3 ERG was measured between its first negative trough and the first positive peak. The amplitude of the PhNR was measured between the baseline and the most negative trough prior to 100 ms time window. The peak time was measured from the presentation of the stimulus to the trough or peak of the relevant wave (Fig. 1).

### Statistical analysis

Statistical analysis was performed using statistical software (SPSS software v. 21.0). The normality of data was checked by Kolmogorov–Smirnov test. Analysis of variance was used to compare electrophysiological parameters between the two groups of subjects. Pearson’s coefficient was used to correlate between parameter amplitudes and between parameter peak time. Partial correlation analysis was used to examine the association between electrophysiological parameters and AL and between the electrophysiological parameters and ages. A *p*-value < 0.05 was considered statistically significant.

## Results

### General characteristics

**Fig. 2:**
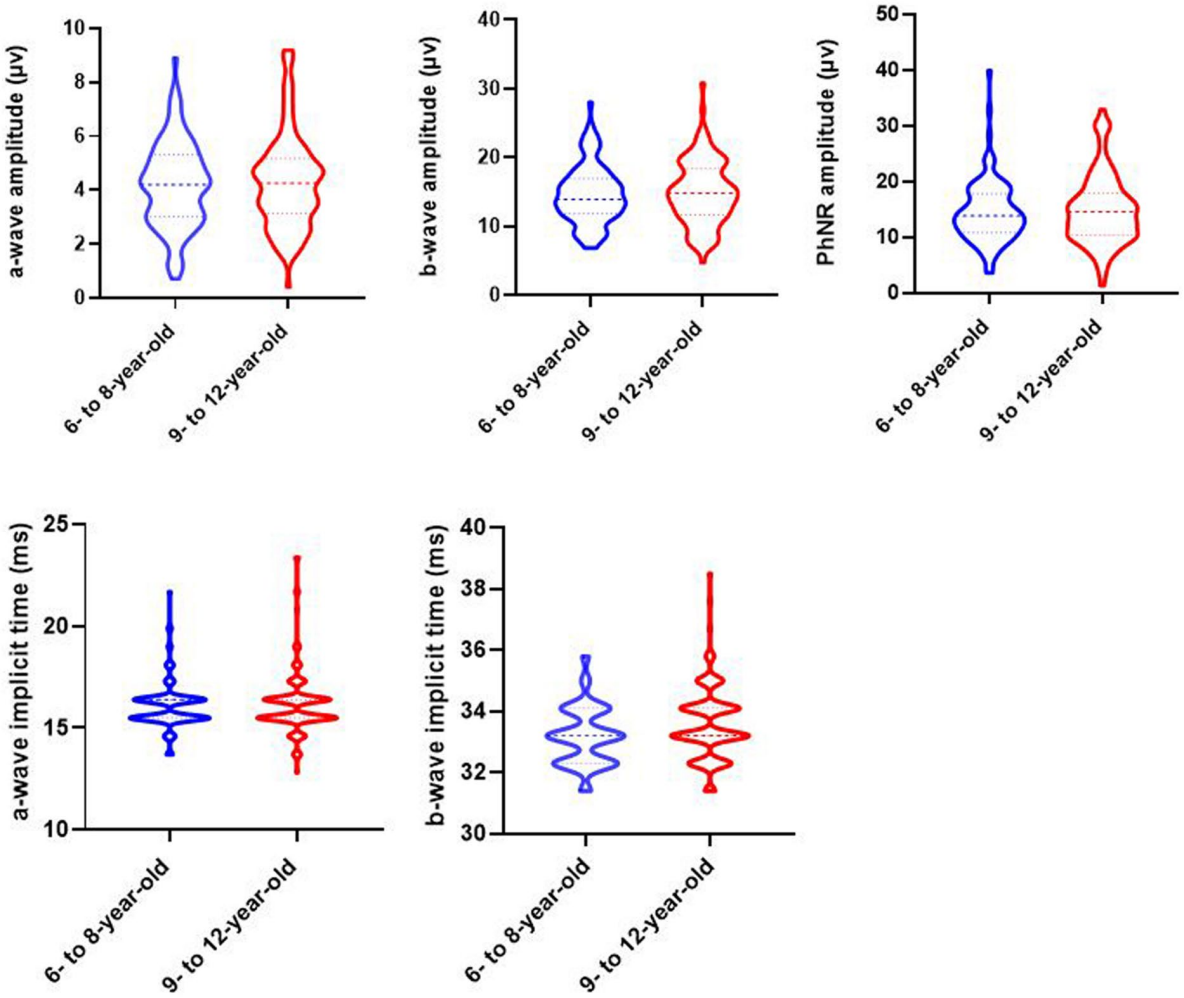
Values of LA 3 ERG and the photopic negative response (PhNR) between two age group in healthy children

A total of 214 eyes of 214 children were enrolled in this study (Fig. 2). The mean age was 8.81 ± 1.70 years old (range, 6–12 years old). The gender distribution was 43.6% boys and 56.4% girls. The median logarithm of the minimal angle resolution best-corrected visual acuity in all patients was 0.00°. Demographics and ocular parameters are shown in Table 1.

**Table 1:** Demographics and ocular parameters for the two groups

Age group (years)	n	Sex girls/boys	Age	Spherical Equivalent	Axial length (mm)
6–8	101	57/44	7.34 ± 0.74	−1.35 ± 0.96	22.89 ± 4.73
9–12	113	61/52	10.13 ± 1.13	−1.59 ± 0.87	22.78 ± 5.93

Data are presented as mean ± standard deviation

### Reference values of LA 3 ERG and PhNR

The reference values for the a-wave and b-wave of the LA 3 ERG and the PhNR amplitudes and peak time are shown in Table 2. 214 eyes for LA 3 ERG and 206 eyes for PhNR, because 8 eyes failed to obtain the true PhNR.

**Table 2:** Reference values of the LA 3 ERG and photopic negative responses

ERG parameters		Amplitude (μV)				Peak time (ms)			
		Minimum	Maximum	Mean ± Std	P2.5, n P97.5	Minimum	Maximum	Mean ± Std	P2.5, n P97.5
LA 3 ERG	a-wave	0.40	9.20	4.25 ± 1.65	1.27, 214 8.56	12.80	23.40	16.20 ± 1.48	13.7, 214 21.39
	b-wave	4.70	30.80	14.78 ± 4.43	7.24, 214 23.73	31.40	38.50	33.41 ± 1.12	31.4, 214 35.8
Photopic negative response	PhNR	1.30	39.90	13.90 ± 5.94	4.46, 206 30.32	–	–	–	–

### ERG characteristics according to age

The group-averaged amplitude and peak time data according to age are shown in Table 3 and Fig. 2. There was a significant difference in the peak time of the b-wave (*p* = 0.01) between the two groups, but no differences in any other parameters. We investigated correlations between each ERG parameter and age using age as a continuous variable. Unfortunately, there were no correlations between each ERG parameter and age (a-wave: amplitude *r* = 0.01, *P* = 0.90, peak time *r* = 0.09, *P* = 0.21; b-wave: amplitude *r* =  − 0.06, *P* = 0.40, peak time *r* = 0.16, *P* = 0.02; PhNR: amplitude *r* =  − 0.06, *P* = 0.40).

**Table 3:** Group-averaged values of measurements for individual LA 3 ERG and photopic negative response amplitudes (μV) and peak time (ms)

Age group (years)		a-Wave			b-Wave			PhNR
	*n*	Amplitude (μV)	Peak time (ms)	*n*	Amplitude (μV)	Peak time (ms)	*n*	Amplitude (μV)
6–8	101	4.19 ± 1.57	16.13 ± 1.22	101	14.63 ± 4.21	33.20 ± 0.96	97	14.50 ± 5.61
9–12	113	4.30 ± 1.73	16.25 ± 1.69	113	14.90 ± 4.62	33.60 ± 1.22	109	14.90 ± 6.24
*p*		0.63	0.56		0.65	0.01		0.63

*p* comparison between the 6- to 8-year-old and 9- to 12-year-old groups

### Correlations between the a-wave, b-wave, and PhNR parameters

**Fig. 3:**
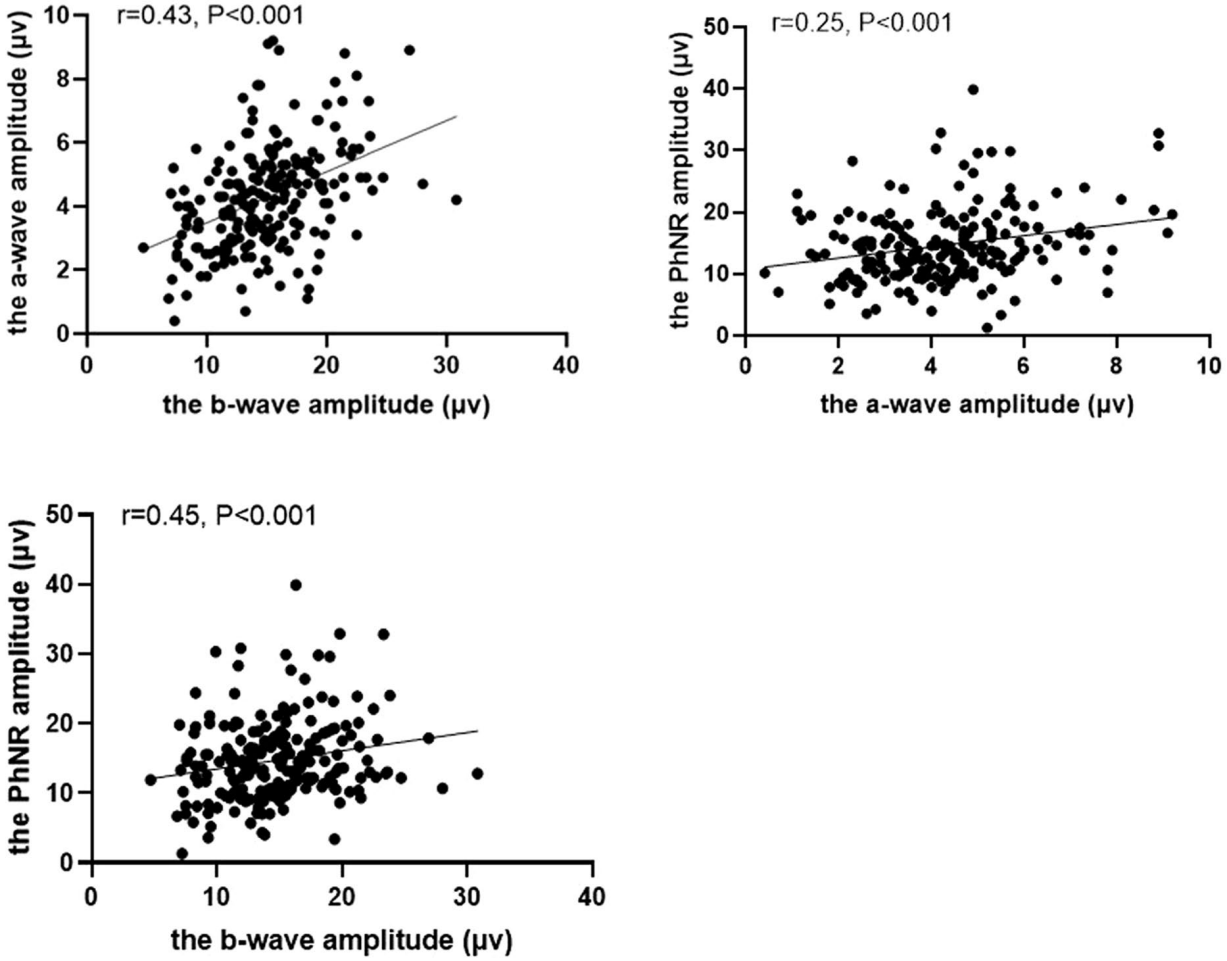
Correlations between the amplitudes of the a-wave, b-wave, and PhNR in healthy children

**Fig. 4:**
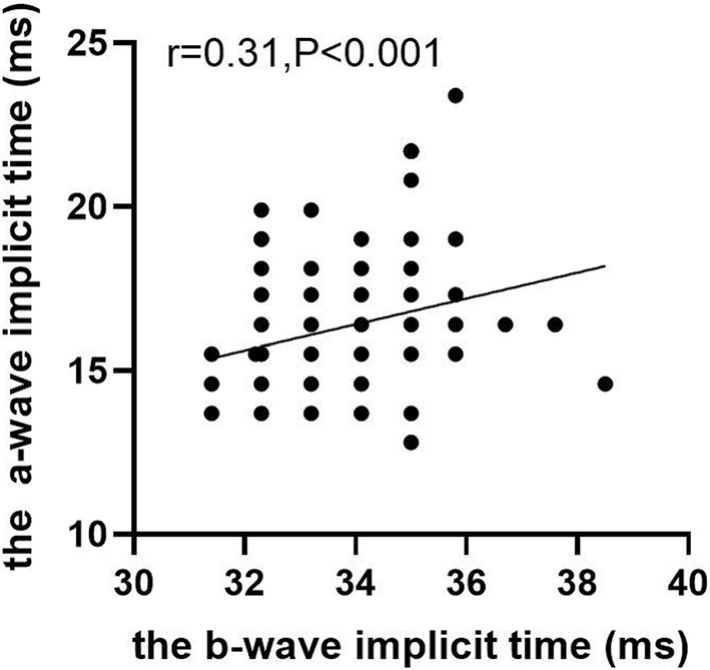
Correlation between the peak time of the a-wave and b-wave in healthy children

The correlations between the electrophysiological parameters in normal children are shown in Figs. 3 and 4. There was a moderate correlation between the a-wave and b-wave amplitudes (*r* = 0.43, *p* < 0.001). There was also a mild correlation between the amplitudes of the a-wave and the PhNR (*r* = 0.25, *p* < 0.001) and a moderate correlation between the b-wave and the PhNR (*r* = 0.45, *p* < 0.001). There was a moderate correlation between the a-wave and b-wave peak time (*r* = *0.31,P* < *0.001*).

### Correlations between electrophysiological parameters and AL

There were no correlations between the electrophysiological parameters and AL (amplitudes a-wave: *r* = 0.06, *p* = 0.42; b-wave: *r* = 0.03, *p* = 0.71; PhNR: *r* = 0.13, *p* = 0.07; peak time a-wave:* r* = *0.05, P* = *0.47*; b-wave:* r* = *0.09, P* = *0.20*).

## Discussion

Changes in the ffERG have been reported in children with various retinal disorders, including Stargardt disease [27] and retinopathy of prematurity [28]. However, the normal ffERG values for children in those studies were derived from healthy control groups with a small sample size and a wide age range. ERG is also affected by the type of electrophysiological recording system, subject age, light stimulation parameters, and electrode type [18, 29–33]. Therefore, in the present study, we determined the normal values of LA 3 ERG and PhNR in a large cohort of children and also examined whether the a-wave, b-wave, and PhNR changed with age especially in 6- to 12-year-old children. Furthermore, we used skin electrodes, which were reported to provide accurate ffERG recording in clinical practice [7, 34, 35] to improve cooperation in children.

Using the Metrovision electrophysiological system, we recorded LA 3 ERG from 214 healthy subjects (mean age, 8.81 years; range, 6–12 years). The mean peak time in the a-wave (16.20 ms) was similar to that reported by Schwitzer et al. (18.6 ms) [29], Esposito et al. (15.73 ms) [31], and Bhatti et al. (14.3 ms) [36]. Furthermore, the mean peak time in the b-wave (33.41 ms) was similar to that reported by Schwitzer et al. (35.80 ms) [29], Esposito et al. (32.16 ms) [31], Abed et al. (30.29 ms) [18], and Bhatti et al. (29.3 ms) [36]. By contrast, the wave amplitudes in our study were similar with previous studies using skin electrodes and were lower than those previously reported using other electrodes. For example, the mean a-wave amplitude (4.25 μV) was similar with Esposito et al. (6.09 μV) [31] and was lower than that reported by Schwitzer et al. (10.8 μV) [29], Lin et al. (91.4 μV) [30], and Bhatti et al. (22.1 μV) [36]. Similarly, the mean b-wave amplitude (14.78 μV) was similar with Esposito et al. (17.37 μV) [31] and Abed et al. (22.35 μV) [18] and was lower than that reported by Schwitzer et al. (48.0 μV) [29], and Bhatti et al. (95.0 μV) [36]. The amplitude in our study which is lower than that in previous studies reported by Lin et al. and Bhatti et al. significantly, because amplitudes are lower with skin electrodes [34]. We can see that the values of different literatures differ greatly, and their sample size is relatively small except for Bhatti et al. study, and age range is relatively large. The data of our large sample may be able to somehow correct bias caused by small samples.

The amplitude of the PhNR (13.90 μV) in the present study was higher than that reported by Mortlock et al. (11.43 μV) [32], Esposito et al. (9.40 μV) [31] and was lower than Abed et al. (19.17 μV) [18] using the same types of electrodes. These differences may relate to differences in the light stimulation parameters and subject age. The amplitudes in all of these studies, including ours, were lower than those reported by Banerjee et al. [33] and Bhatti et al. [36], which likely relates to the different electrodes used in that study.

In the present study, there was only a small difference in the peak time of the b-wave between the 6- to 8-year-old and the 9- to 12-year-old groups, which may be of limited clinical significance, while there were no differences in any other parameters. This indicates that the electrophysiology of retinal cells in school-age children does not change with age. It is also possible that because the amplitude obtained by the skin electrode is small, the correlation is not easily detected.

The significant correlation between the amplitudes of the a-wave and b-wave in LA 3 ERG was consistent with the findings of Esposito et al.[31], which confirms the reliability and repeatability of our study. Skin electrodes placed farther from the eyelid margin can reduce amplitude responses, though less effect on peak time [37]. In order to be more reflective of retinal neuronal processing times, we also analyzed correlation between the peak time of the a-wave and b-wave. The significant correlation between the a-wave and b-wave peak time also existed. These findings suggest the existence of a strict functional relationship between cone cell pathway components in the eye [31]. We also found a positive correlation between PhNR amplitude and the a-wave and b-wave amplitudes, as reported by Esposito et al. [31], suggesting that ganglion cell function is affected by more distal retinal elements in the eye. In addition, the farther the active electrode is from the eyelid margin, the lower the recorded signal amplitude [37, 38]. So, we made sure that our electrode positioning is consistent with Esposito et al. However, the extent to the amplitude of each wave reduction remains unclear. So, the influence of electrode position on correlation needs to be further explored.

By contrast, we found no correlation between the electrophysiological parameters and AL. Previous studies have reported a reduction in electrophysiological amplitudes in high myopia and pathological myopia patients [39, 40]. In adults, axial elongation of the myopic eye can stretch the retina across the interior of the globe, thereby reducing the sampling density of retinal neurons and altering retinal physiology [41]. Previous study has reported peak time showed minimal delay with increase in axial length in adults. However, this has not yet been reported in children. Additionally, the present study only included children with a refractive error <  ± 6.00 D mean sphere, which resulted in a small AL range (21.37–26.32 mm). Thus, we did not find the same relationship as observed in adults. Further studies are required in children with a larger AL range.

There are some limitations to our study. First, the electrophysiological examination was not repeated for every child, although only stable electrophysiological results from cooperative children were selected for analysis. Second, because we used skin electrodes, the ffERG values are not comparable to studies using corneal electrodes. However, skin ERG electrodes can facilitate better testing cooperation in children. Thirdly, we recorded only LA 3 ERG and PhNR. However, in order to record the ERG responses for the younger children, who are not able to cooperate with the longer examination, we could not record other 5 standard ffERG responses. Due to the difficulty of examining children, and to ensure the reliability of the data, most of the published papers did not report records including 6 standard full-field ERG responses. Although establishing laboratory-specific reference values is the most optimal process, now lacking large sample electrophysiological data in children as a basic reference, we recorded a large number of electrophysiological results in 6- to 12-year-old children, especially including PhNR, which can at least provide a reference value for public. Fourthly, our study filtered to a much narrower range of frequencies than is conventional, but the ffERG results obtained in the 1–35 Hz range were more stable and reproducible in our laboratory with reference to previous research [42]. In addition, we detected ffERG following ISCEV standard protocols as strictly as possible.

To our knowledge, this study provides the largest dataset of LA 3 ERG and PhNR parameters in a population of healthy children. These electrophysiological parameters may be useful reference values when evaluating children with potential retinal defects. However, these results should be interpreted with caution because of different skin electrodes and narrower range of frequencies.

## Declarations

### Statement on the welfare of animals

No animals were used in this research.
